# Supplementary material for: Mini-Intein Structures from Extremophiles Suggest a Strategy for Finding Novel Robust Inteins
Source: Microorganisms. 2021 Jun 5;9(6):1226. doi: 10.3390/microorganisms9061226 (PMC8229266; doi:10.3390/microorganisms9061226)
Supplement: Supplementary file 1 [file microorganisms-09-01226-s001.zip › microorganisms-1231395-supplementary.pdf]

Table S1: A list of protein hosts containing inteins in the four thermophiles

**Protein hosts**		***Mja***	***Pho***	***Pfu***	***Pab***
Vacuolar-type ATPase, catalytic subunit		–	376	425	429
Translation initiation factor biF-2		546	444	387	394
KlbA virulence protein		**168**	520	522	**196**
Uncharacterized protein (*E. coli* rtcB homolog)		488	390	380	436
Replication factor C, 37 kD subunit	a	548	525	525	499
	b	436	–	–	–
	c	543	–	–	608
DNA reverse gyrase/DNA topoisomerase I		494	410	373	–
Archaeal type B DNA polymerase Pol I	a	369	–	–	–
	b	476	460	–	–
Archaeal DNA polymerase Pol II, DP2 subunit		–	**166**	–	**185**
Cell division control protein 21 (CDC21)	a	–	**168**	–	**164**
	b	–	260	367	268
ATP-dependent protease LA (lon)		–	474	401	333
Class-II Ribonucleotide reductase	a	×	–	454	399
	b	×	385	382	382
	c	×	–	–	438
Class-III Ribonucleotide reductase	a	453	–	–	–
	b	533	–	–	–
DNA binding protein radA		–	**172**	–	–
LHR-large helicase-related protein		–	475	–	–
DEAD/DEAH-box helicase		501	–	–	–
RNA polymerase subunit A′		452	–	–	–
RNA polymerase subunit A″		471	–	–	–
Transcription factor IIB		335	–	–	–
Phosphoenol pyruvate synthase		412	–	–	–
UDP-glucose dehydrogenase		454	–	–	–
Uncharacterized protein (Mja0043)		392	–	–	–
Glutamine fructose-6-phosphate transaminase		499	–	–	–
Molybdenum cofactor biosynthesis protein A		–	–	–	455
**Total number of inteins**		19	14	10	14

Mja, *Methanococcus jannaschii*; ***Pho***, *Pyrococcus horikoshii*; ***Pfu***, Pyrococcus furiosus; ***Pab***, *Pyrococcus abyssi*. The numbers indicate the sizes of inserted inteins by residues. Mini-inteins are highlighted in yellow
